# Nutritional Content and Health Profile of Single-Serve Non-Dairy Plant-Based Beverages

**DOI:** 10.3390/nu14010162

**Published:** 2021-12-30

**Authors:** Winston J. Craig, Cecilia J. Brothers, Reed Mangels

**Affiliations:** 1 Center for Nutrition, Healthy Lifestyles, and Disease Prevention, School of Public Health, Loma Linda University, Loma Linda, CA 92354, USA; 2 Department of Biology, Walla Walla University, College Place, WA 99324, USA; cecilia.brothers@wallawalla.edu; 3 Vegetarian Resource Group, Baltimore, MD 21203, USA; reedmangels@gmail.com

**Keywords:** non-dairy milk alternatives, plant-based beverages, protein, calcium, vitamin D, vitamin B12, sugar, single-serve beverages, school meal programs

## Abstract

A growing number of people are seeking a non-dairy plant-based beverage both for their personal health, and for the health of the planet. The aim of this study was to conduct a cross-sectional survey of single-serve plant-based beverages to assess their nutritional content and health profile. A total of 51 non-dairy plant-based beverages were analyzed from the nutrition label listed on the commercial package. The various beverages contained extracts of soy (*n* = 14), almonds (*n* = 13), oats (*n* = 12), peas (*n* = 7), banana (*n* = 2), coconut (*n* = 2), and rice (*n* = 1). Almost one-half (45%) of the single-serve beverages had 5 g or more of protein/serving. A total of 75% and 65% of the single-serve beverages had calcium and vitamin B12 levels, respectively, fortified to at least 20% of the Daily Value (DV), while only 28% had vitamin D fortification at the 20% DV level. Two-thirds of the single-serve beverages had high sugar levels, while 39% were low in sodium, 63% were low in fat, and 96% were low in saturated fat. The single-serve plant-based beverages had more protein, calcium, vitamin B12, and sugar but less fat than the non-dairy, multi-serve plant-based beverages/ serving. A limited number of single-serve beverages met the requirements of school meal programs.

## 1. Introduction

Non-dairy plant-based beverages, also known as plant milks, are an increasingly popular product category. These beverages are based on soy, nuts, seeds, grains, or other ingredients. They may be chosen by those with milk protein allergy or lactose intolerance by those who are limiting dairy consumption due to health, environmental or animal welfare concerns, or by those simply wanting to try different products [1]. In the United States, retail sales of plant-based milk increased 20% in 2020, the most recent year for which sales data are available [2]. Plant-based milks are purchased by 39% of U.S. households [2]. These beverages are often fortified with nutrients including calcium, vitamin D, and vitamin B12, although fortification levels vary [3]. Positive features of many products include low levels of saturated fat, sodium, and sugars; some products also supply a significant amount of protein and some fiber [3].

In the United States, non-dairy plant-based beverages are commonly packaged in multi-serving containers, often 64 fluid oz (1.89 L), 48 fluid oz (1.42 L), or 28 fluid oz (828 mL) refrigerated containers. Shelf-stable aseptic packages are typically 33.8 fl. oz (1.0 L) or 32 fl. oz (946 mL). Products are also available in single-serving packages, which range from 200 mL to 355 mL. These single-serving packages offer convenience and may be used by those consuming meals away from home, while traveling, or in restaurants and hospitals. They may be included in the lunches children bring from home to school or daycare. Fortified soy or pea-protein-based beverages are recommended as a primary beverage for young children who do not use dairy milk, while older children can use other fortified plant beverages such as those based on almonds or oats [4].

Schools, daycare homes, and daycare centers in the United States that participate in the National School Lunch Program (NSLP), the National School Breakfast Program (NSBP), or the Child and Adult Care Food Program (CACFP) may choose to offer one or more substitutes for fluid dairy milk for non-disabled children with medical or special dietary needs [5]. These substitutes must meet standards set by the USDA. Namely, an 8-ounce serving is required to contain at least 8 g protein, 276 mg calcium, 500 IU vitamin A, 100 IU vitamin D, 24 mg magnesium, 222 mg phosphorus, 349 mg potassium, 0.44 mg riboflavin, and 1.1 mcg vitamin B12 [5]. Optional best practices for programs in the CACFP include serving only unflavored milks [6]. Some single-serve products may meet the requirements for use in these programs.

The objectives of this study are to assess the nutritional content of single-serve non-dairy plant-based beverages and to compare these products to products providing multiple servings. We wanted to find out if single-serve products, a portion of whose market may be households with children, would differ in terms of nutritional content from multi-serving products. We also sought to determine if some single-serve products met the requirements to be used as fluid milk substitutes in the NSLP, the NSBP, and the CACFP. These comparisons will be useful when professionals and consumers make decisions about the use of these single-serve products.

## 2. Materials and Methods

The nutritional contents of 51 single-serve plant-based non-dairy beverages, representing 16 brands, were recorded from the nutrition label on the commercial package. The single-serve beverages were selected from February to April 2021 from those available in supermarkets and stores in the western USA. Additional varieties of plant-based non-dairy beverages were analyzed from the nutritional labels given by the manufacturer’s website or from the website of common retailers. No smoothies or protein shakes were included in the analysis. The nutrients per serving size, which were available on all packages, included calories, fat, saturated fat, sodium, carbohydrates, dietary fiber, total sugars, protein, and the micronutrients calcium, vitamin D, and vitamin B12. The median values of the nutrients were calculated for each type of beverage base. The values for each nutrient were compared amongst the 5 different bases.

A similar analysis was conducted on 323 multi-serve plant-based non-dairy beverages available in the USA, thus that a comparison could be made between the nutritional value of 1 serving of the multi-serve plant-based beverage (non-dairy alternative) with that of 1 serving of the single-serve plant-based beverage. The levels of fortification for calcium, vitamin D, and vitamin B12 were calculated for all beverages.

The US Dietary Guidelines specify, as a general guide, that 5% DV or less of a nutrient/serving was considered low, while 20% DV or more of a nutrient/serving was considered high [7,8]. The nutritional value of each plant-based non-dairy beverage was rated according to the following criterion: calcium, vitamin D, and vitamin B12 of at least 20% of Daily Value (DV)/serving; and at least 5 g of protein/serving (10% of the DV). The health qualities demonstrated by the ingredients were determined by the following criteria: not more than 5 g of total sugars/serving (10% of DV); less than 4 g fat/ serving (5% DV); not more than 1 g of saturated fat/serving (5% of the DV); not more than 140 calories/serving; not more than 115 mg sodium/serving (5% of DV); and at least 1.5 g (10% of DV) of dietary fiber/serving. In the USA the DV for calcium is 1300 mg, vitamin D is 20 mcg, vitamin B12 is 2.4 mcg, sodium is 2300 mg, protein is 50 g, added sugars is 50 g, saturated fat is 20 g, and dietary fiber is 28 g [8]. A total of 10% was chosen as a mid-stream number between the 5% DV (low value) and the 20% DV (high value) for protein and sugars. For calculations, total sugars were used throughout since added sugars, and total sugars were essentially the same.

### Statistical Analysis

R software was used to conduct all statistical analyses [9]. Data were tested for normality and homoscedasticity prior to analysis. The median and interquartile ranges were used for descriptive statistics, as the data were not normally distributed. The nutritional content was compared across the different bases of non-dairy single-serve beverages using a Kruskal–Wallis test for each nutrient, followed by Dunn’s posthoc test with Bonferroni adjustment for multiple comparisons. The nutritional content was analyzed between non-dairy single-serve beverages and multi-serve beverages using an unpaired Wilcoxon rank-sum test for each nutrient. A significant *p*-value of less than 0.05 was used for all analyses.

## 3. Results

The 51 plant-based non-dairy beverages analyzed were based upon soy (*n* = 14), almonds (*n* = 13), oats (*n* = 12), pea protein (*n* = 4), banana (*n* = 2), coconut (*n* = 2), flax with pea protein (*n* = 2), rice (*n* = 1), almond with pea protein (*n* = 1). Table 1 displays the medians of each nutrient for all of these base types, and significant differences among the base types are reported.

One serving size of the single-serve beverage varied according to the size of the container. The most common size was 240 mls (*n* = 38; 74.5%). Other sizes included 200 mls (*n* = 2), 295 mls (*n* = 2), 330 mls (*n* = 4), and 355 mls (*n* = 5). Fourteen of 16 brands had 1 to 4 varieties each, while the remaining two brands had 8 to 9 varieties each, thus representing one-third of all the beverages. 

Nineteen (37.3%) of the single-serve beverages were chocolate flavored, 14 (27.5%) were vanilla flavored, 17 (33.3%) were not flavored (plain), and one (2.0%) was blueberry flavored. Five (9.8%) were labeled as unsweetened. Four beverages (7.8%), all of one brand, had no fortification whatsoever. Table 2 outlines the number of single-serve non-dairy beverages that are fortified with calcium, vitamin D, and/or vitamin B12. Tricalcium phosphate (TCP) (43%), calcium carbonate (28%), and a TCP-calcium carbonate mix (10%) were the most commonly used calcium salts. About 14% had no added calcium. Gellan gum (61%) was the most commonly added fiber. Other fibers added to the beverages included locust bean gum (22%), xanthan gum (18%), carrageenan (18%), and guar gum (12%). One beverage contained tara gum, and another contained the prebiotic fiber inulin. Two beverages contained monk fruit, and one had Reb A for sweetening.

Table 3 summarizes the data showing the percentage of the single-serve non-dairy beverages that meet the suggested nutrient guidelines per serving, including those with low levels of sugar, sodium, and saturated fat. The 323 multi-serve plant-based beverage alternatives analyzed were based upon almonds (*n* = 83), oats (*n* = 62), soy (*n* = 44), coconut (*n* = 24), hemp (*n* = 15), pea protein (*n* = 11), rice (*n* = 11), cashews (*n* = 10), flax (*n* = 6), banana (*n* = 6), macadamia (*n* = 6), hazelnuts (*n* = 4), chia (*n* = 4), quinoa (*n* = 2), pili nut (*n* = 2), walnut (*n* = 1), pistachio (*n* = 1), and the following mixtures: almond and pea (*n* = 7), almond and coconut (*n* = 5), sesame and pea (*n* = 5), flax and pea (*n* = 4), oats and pea (*n* = 2), oats and avocado (*n* = 2), rice and quinoa (*n* = 2), almond and cashew (*n* = 2), coconut, cashew, and oats (*n* = 1), and almond and sesame (*n* = 1) [10]. For the multi-serve plant-based beverage alternatives, 121 (37.5%) were unsweetened. While 60% of the plant-based beverage alternatives were plain, the most common flavors were vanilla (25%) and chocolate (9%). The typical serving size of the beverage was 240 mls.

Table 4 compares the nutrient composition of single-serve plant-based beverages with multi-serve plant-based beverages. Significant differences were noted for 8 of the 11 nutrients. Only saturated fat, sodium, and vitamin D were the exceptions. Forty single-serve beverages representing 14 brands could be matched with multi-serve beverages of the same brand, base, and flavor. Of these 40 single-serve beverages, 27 had the same or similar nutrient levels as the multi-serve beverages, while 3 were sweeter by 6–8 g sugar/serving. Of the 10 single-serve beverages with fortification patterns different from the multi-serve, 4 had adequate B12 levels (versus none in the multi-serve), while 3 single-serve had zero vitamin D (versus adequate levels in the multi-serve). While 37.5% of the multi-serve beverages were unsweetened, only 10% of the single-serves were unsweetened. The chocolate flavor was more common among the single-serve (37%) than the multi-serve beverages (9%).

## 4. Discussion

Non-dairy, plant-based beverages are increasing in popularity. Concerns exist regarding their nutritional value, especially with respect to the level of protein and the level of fortification of calcium, vitamin D, and vitamin B12. Because the single-serve beverages are utilized in schools, daycare homes, and daycare centers, these nutritional concerns take on extra importance since there are nutrition standards that must be met by the food manufacturers for their products. The median values of nutrients in the 51 single-serve, non-dairy plant-based beverages, reported by base type in Table 1, show that those beverages based upon a legume (soy or pea protein) had protein levels/serving similar to that of dairy milk (see Table S1). A large majority of the beverages had calcium fortification, similar to calcium levels in dairy beverages. Many of the beverages, except those based upon almonds, had adequate vitamin B12 fortification levels (45–50% of DV). A total of 84% of the beverages had vitamin D fortification, with median levels across the base types of 10–15% of DV (compared to dairy vitamin D levels of 14% DV, see Table S1). 

Since the NSLP/NSBP/CACFP standards require non-dairy plant-based beverages to have at least 8 g of protein, only those based on soy or pea protein qualify. Furthermore, both soy protein [11] and pea protein [12] have amino acid profiles that make them a good quality protein source.

While the single-serve beverages that were calcium-fortified generally had 15–40% of the calcium DV, 4 brands had zero calcium fortification. These beverages were largely based on bananas, oats, or almonds. Calcium fortification was primarily achieved with 2 different salts, tricalcium phosphate (TCP) and calcium carbonate. The absorbability of calcium varies depending upon the food matrix, including the presence of stabilizers [13,14]. Calcium carbonate absorption is very similar to milk calcium (which has a fractional absorption of about 30%), while the absorption of TCP is somewhat less than milk calcium [13]. 

How does a serving of a non-dairy plant-based beverage compare to that of a non-dairy yogurt alternative when it comes to supplying the essential vitamins D and B12 for people who follow a vegan diet? The incidence of vitamin D and B12 fortification was 2 to 3 times greater than those found in non-dairy yogurt alternatives [10], while the median level of vitamin B12 in the single-serve beverages was twice that of the non-dairy yogurt alternatives. The vitamin B12 fortification was poorest in almond–based beverages, with 70% having no fortification at all. 

The median vitamin D level of the legume-based beverages tended to be higher than that of oat- and almond-based beverages (Table 1). Eight beverages had zero vitamin D fortification, 4 almond beverages of one brand, and 4 oat beverages of a second brand. Non-dairy beverages are typically fortified with ergocalciferol (vitamin D2 rather than vitamin D3 since the latter is normally derived from animal sources and hence unacceptable to people who follow vegan diets. Although D3 is better absorbed than D2, both forms of vitamin D in fortified foods are well absorbed in the small intestine [15] and are effective in increasing total 25-hydroxyvitamin D levels [16].

### 4.1. Health Profile

Consumers, for health reasons, are often concerned about the level of sodium (salt), saturated fat, and sugars in their food. An excess of any of these 3 nutrients can have negative health effects [17,18,19,20,21]. Almost 40% of the single-serve plant-based beverages had low levels of sodium, and 63% had low levels of fat, while 62% had high levels of sugar (at least 10 g/serving) (Table 3). Most of the beverages (96%) had low levels of saturated fat. The median level of saturated fat for the plant-based beverages was 0.5 mg. This compares with the typical value of 5.1 g for whole dairy milk and 2.9 g of saturated fat for low-fat (2%) dairy milk [22]. The single-serve plant-based beverages that are legume-based tended to have lower levels of sodium than the almond-based beverages, while they tended to have higher levels of sugar than the oat-based and higher levels of fat than the almond- and oat-based beverages (Table 1). The mean caloric content of the soy- and oat-based beverages was twice that of the almond-based beverages per serving. The mean caloric content of the soy- and oat-based beverages was similar to that of whole cow’s milk but lower than low-fat chocolate milk [22]. Only 14% of the single-serve beverages contained over 150 calories/serving, and the majority were chocolate flavored. The chocolate-flavored beverages contained the highest levels of sugar.

Almost one-half of the single-serve beverages had at least 1.5 g fiber/serving (Table 2). A variety of water-soluble fibers (gums) were used as emulsifiers and thickening agents, with gellan gum being the most commonly used. Other fibers included locust bean gum, xanthan gum, carrageenan, and guar gum. The gums can help influence glycemia and hypercholesterolemia [23,24,25].

An emerging health issue is a concern about estradiol in cow milk. Milk consumption can increase serum estradiol levels [26]. The use of dairy milk was found in the Adventist Health Study-2 cohort to be positively associated with the risk of breast cancer [27]. The plant-based milk alternatives do not contain estradiol. While the soy-based milk alternatives contain isoflavones, classified as phytoestrogens, soy products have no negative effects in children. An extensive review [28] suggested that neither isoflavones nor soy foods could be classified as endocrine disruptors associated with adverse health outcomes such as breast cancer.

### 4.2. Comparison to Beverages

The single-serve beverages had significantly higher median values for protein, calcium, and vitamin B12 than the overall multi-serve beverages (Table 4). The higher levels of sugar in the single-serve beverages may reflect the fact that fewer were labeled as unsweetened (10% versus 37.5%) and 4 times as many were chocolate-flavored. From the comparison in Table 4, the single-serve beverages typically have a better nutritional profile than the multi-serve beverages as a whole. The single-serve beverages, which had matching multi-serve beverages of the same brand, base, and flavor, were relatively comparable. Generally, the food manufacturers have selected beverages with a better nutritional profile to deliver in the single-serve format.

Single-serve beverages are a convenient alternative to multi-serve beverages in school meals programs, but many do not appear to meet the requirements of these programs. Of the 51 single-serve beverages evaluated, 9 (18%) appeared to meet the requirements for the NSLP, NSBP, and CACFP. The most common shortfall was protein. Greater attention to these requirements by food manufacturers would allow additional products to be used in Child Nutrition Programs. In addition to potentially being used by school meal programs, single-serve beverages are used in school meals that are brought from home. Some, but not all, products meet suggested nutrient guidelines (Table 3). Parents and caregivers should be encouraged to select the most nutritious products.

A strength of this study is its detailed evaluation of single-serve non-dairy plant-based beverages available in the United States. Product formulations change, thus, this study reflects product composition at one point in time. Information was obtained from package labels, manufacturers’ websites, and common retailers’ websites and not from direct analysis. This package and website information is what is used by consumers use in making purchase decisions.

## 5. Conclusions

While non-dairy plant-based beverages are increasingly popular, single-serve beverages have a more targeted consumer audience. A number of these single-serve beverages have been developed for special populations such as school children and child and adult care centers. It is important that we analyze these beverages to assess their nutritional content and health profile. The single-serve beverages are largely based upon 4 different plant foods—soy, oats, almonds, and peas. Almost one-half (45%) of the single-serve beverages had 5 g or more of protein/serving, while 75% and 65% of the beverages had calcium and vitamin B12 levels, respectively, fortified to at least 20% of DV. However, only 28% had vitamin D fortification at the 20% DV level. Two-thirds of the single-serve beverages had high sugar levels, while 39% were low in sodium, 63% were low in fat, and 96% were low in saturated fat. The single-serve plant-based beverages had more protein, calcium, vitamin B12, and more sugar but less fat than the non-dairy, multi-serve plant-based beverages/serving. A limited number of single-serve beverages met the requirements of school meal programs.

## Figures and Tables

**Table 1:** Median (Q1–Q3) values of nutrients in non-dairy single-serve beverages/serving for all base types.

Nutrient	All	Soy	Almond	Oat	Pea/Pea Blend	Others ^1^	*p*
*n*	51	14	13	12	7	5	
Calories	120 (90–140)	140 (130–150) ^bc^	70 (40–90) ^a^	145 (130–170) ^b^	120 (100–140) ^abc^	90 (80–90) ^ac^	<0.001
Fat (g)	3 (2.5–4.5)	4.5 (3.6–4.5)	2.5 (2.5–3)	2.75 (2.5–4.6)	4.5 (3.3–4.5)	2.5 (0–4)	0.026
Satd. fat (g)	0.5 (0–0.5)	0.5 (0.5–0.9) ^b^	0 (0–0) ^a^	0.5 (0.4–0.5) ^b^	0.5 (0–0.5) ^ab^	0 (0–4) ^ab^	0.003
Sodium (mg)	125 (97.5–160)	110 (86.3–123.8) ^b^	170 (150–180) ^a^	122.5 (107.5–157.5) ^ab^	110 (97.5–135) ^ab^	90 (90–100) ^b^	<0.001
Carbohydrates (g)	17 (12.5–22)	16.5 (13.3–18) ^ab^	11 (2–19) ^a^	23.5 (20.5–27.3) ^b^	12 (11–15.5) ^a^	19 (13–22) ^ab^	<0.001
Fiber (g)	1 (0.7–2)	2 (1–2) ^ab^	1 (0.3–1) ^a^	3 (2–3) ^b^	1 (0.25–1.5) ^ab^	1 (0–2) ^ab^	<0.001
Sugars (g)	10 (7–15)	12 (10.3–15)	10 (0–17)	7 (6–10)	11 (9–14)	10 (10–12)	0.245
Protein (g)	4 (1–8)	8 (6.3–8) ^b^	1 (1–1) ^a^	4 (3–4) ^ab^	8 (7.5–8) ^b^	1 (1–1) ^a^	<0.001
Calcium (%DV)	30 (17.5–35)	30 (25–35)	30 (4–35)	25 (2.8–35)	30 (22.5–35)	4 (0–10)	0.097
Vit. D (%DV)	10 (10–20)	15 (15–15)	10 (0–10)	10 (0–25)	15 (10–30)	20 (20–25)	0.004
Vit. B12 (%DV)	40 (0–50)	45 (5–90)	0 (0–25)	40 (0–80)	45 (45–50)	50 (25–60)	0.044

Different superscript letters (a, b, c) in the same row indicate significant differences among base types. *p* < 0.05 is considered statistically significant. “All” was not included in the analyses. ^1^ Banana (*n* = 2), coconut (*n* = 2), rice (*n* = 1).

**Table 2:** Number (%) of non-dairy beverages that are fortified with calcium, vitamin D, and vitamin B12.

Nutrient	Single-Serve Beverages	Multi-Serve Beverages
*n*	51	323
Calcium	40 (78.4%)	236 (73.1%)
Vit. D	43 (84.3%)	226 (70.0%)
Vit. B12	33 (64.7%)	135 (41.8%)

**Table 3:** Number (%) of non-dairy beverages meeting the suggested nutrient guideline per serving.

Nutrient	Single-Serve Beverages	Multi-Serve Beverages
*n*	51	323
At least 20% DV of Calcium	38 (74.5%)	213 (65.9%)
At least 20% DV of vitamin D	14 (27.5%)	133 (41.1%)
At least 20% DV of vitamin B12	33 (64.7%)	133 (41.1%)
At least 5 g protein (10% DV)	23 (45.1%)	70 (21.7%)
No more than 115 mg sodium (5% DV)	20 (39.2%)	175 (54.2%)
No more than 1 g saturated fat	49 (96.1%)	289 (89.4%)
Less than 4 g fat (5% DV)	32 (62.7%)	160 (49.5%)
No more than 140 calories	16 (31.4%)	305 (94.4%)
At least 1.5 g fiber	23 (45.1%)	78 (24.1%)
10 g or more of total sugars	32 (62.7%)	68 (21.1%)

**Table 4:** Median (Q1–Q3) values for nutrients in single-serve non-dairy beverages contrasted with that of one serving of multi-serve non-dairy beverages.

Nutrient	Single-Serve Beverages	Multi-Serve Beverages
*n*	51	323
Calories	120 (90–140) ^a^	80 (55–120) ^b^
Fat (g)	3 (2.5–4.5) ^a^	4 (2.5–5) ^b^
Saturated fat (g)	0.5 (0–0.5)	0.5 (0–1)
Sodium (mg)	125 (97.5–160)	110 (90–150)
Carbohydrates (g)	17 (12–22) ^a^	8 (2–15) ^b^
Fiber (g)	1 (0.65–2) ^a^	1 (0–1) ^b^
Sugar (g)	10 (7–15) ^a^	5 (0–8) ^b^
Protein (g)	4 (1–8) ^a^	2 (1–4) ^b^
Calcium (% DV)	30 (17.5–35) ^a^	25 (5–30) ^b^
Vitamin D (% DV)	10 (10–20)	15 (0–25)

Different superscript letters (a, b) in the same row indicate a significant difference between beverages. *p* < 0.05 is considered statistically significant.

## Data Availability

Not applicable.

## Supplementary Materials

The following is available online at https://www.mdpi.com/article/10.3390/nu14010162/s1. Table S1: Nutrient levels (per 8 oz. serving) in 2% dairy milk, with added vitamins A and D.
