# Supplementary material for: Nutritional Content and Health Profile of Single-Serve Non-Dairy Plant-Based Beverages
Source: Nutrients. 2021 Dec 30;14(1):162. doi: 10.3390/nu14010162 (PMC8747653; doi:10.3390/nu14010162)
Supplement: Supplementary file 1 [file nutrients-14-00162-s001.zip › nutrients-1510129-supplementary.pdf]

Table S1. Nutrient levels (per 8 oz. serving) in 2% dairy milk, with added vitamins A and D

Calories	122
Protein	8.2 g
Total fat	4.66 g
Saturated fat	2.72 g
Sugars	12 g
Calcium	309 mg (24% DV)
Magnesium	29.4 mg
Phosphorus	252 mg
Potassium	390 mg
Sodium	96 mg
Vitamin A	203 mcg
Vitamin B12	1.35 mcg (56% DV)
Vitamin D	2.77 mcg (14% DV)

Source: https://fdc.nal.usda.gov/fdc-app.html#/food-details/746778/nutrients
